# Third-Party Allogeneic Mesenchymal Stromal Cells Prevent Rejection in a Pre-sensitized High-Risk Model of Corneal Transplantation

**DOI:** 10.3389/fimmu.2018.02666

**Published:** 2018-11-20

**Authors:** Paul Lohan, Nick Murphy, Oliver Treacy, Kevin Lynch, Maurice Morcos, Bingling Chen, Aideen E. Ryan, Matthew D. Griffin, Thomas Ritter

**Affiliations:** ^1^Regenerative Medicine Institute, School of Medicine, College of Medicine, Nursing and Health Sciences, National University of Ireland Galway, Galway, Ireland; ^2^Discipline of Pharmacology and Therapeutics, School of Medicine, College of Medicine, Nursing and Health Sciences, National University of Ireland Galway, Galway, Ireland; ^3^CÚRAM Centre for Research in Medical Devices, School of Medicine, College of Medicine, Nursing and Health Sciences, National University of Ireland Galway, Galway, Ireland

**Keywords:** mesenchymal stromal cells, cornea transplantation, high-risk, pre-sensitization, regulatory T cells, immunomodulation, third-party

## Abstract

High-risk cornea transplant recipients represent a patient population with significant un-met medical need for more effective therapies to prevent immunological graft rejection due to heightened anti-donor immune response. In this study, a rat model of pre-existing anti-donor immunity was developed in which corneal allografts were rejected earlier than in non-pre-sensitized recipients. In this model, third-party (non-donor, non-recipient strain) allogeneic mesenchymal stromal cells (allo-MSC) were administered intravenously 7 and 1 days prior to transplantation. Rejection-free graft survival to 30 days post-transplant improved from 0 to 63.6% in MSC-treated compared to vehicle-treated control animals (*p* = < 0.0001). Pre-sensitized animals that received third-party allo-MSC prior to transplantation had significantly higher proportions of CD45^+^CD11b^+^ B220^+^ monocytes in the lungs 24 h after the second MSC injection and significantly higher proportions of CD4^+^ FoxP3^+^ regulatory T cells in the graft-draining lymph nodes at the average day of rejection of control animals. In *in vitro* experiments, third-party allo-MSC polarized primary lung-derived CD11b/c^+^ myeloid cells to a more anti-inflammatory phenotype, as determined by cytokine profile and conferred them with the capacity to suppress T cell activation via prostaglandin E_2_ and TGFβ1. In experiments designed to further validate the clinical potential of the protocol, thawed cryopreserved, third-party allo-MSC were shown to be similarly potent at prolonging rejection-free corneal allograft survival as their freshly-cultured counterparts in the pre-sensitized high-risk model. Furthermore, thawed cryopreserved third-party allo-MSC could be co-administered with mycophenolate mofetil without adversely affecting their immunomodulatory function. In conclusion, a clinically-relevant protocol consisting of two intravenous infusions of third-party allo-MSC during the week prior to transplantation, exerts a potent anti-rejection effect in a pre-sensitized rat model of high-risk corneal allo-transplantation. This immune regulatory effect is likely to be mediated in the immediate post-transplant period through the promotion, by allo-MSC, of alternatively-activated macrophages in the lung and, later, by enhanced regulatory T-cell numbers.

## Introduction

High immunological risk cornea transplant recipients have a much higher rate of rejection and lower long-term graft survival than conventional cornea transplant recipients (1). Factors that place patients at high-risk of rejection include corneal ulcers, bullous keratopathy or a failed previous graft (1, 2). These conditions frequently lead to increased blood and lymphatic vessel infiltration to the recipient graft bed and/or some pre-existing donor-specific cellular immune response (3). As outcomes for lower-risk cornea transplant recipients, such as those receiving a first graft for keratoconus and other non-inflammatory conditions, have been optimized through technical developments, the immunological focus for cornea transplant-related research is shifting toward improving the therapeutic options and outcomes for high-risk patients (3). Pre-clinical high-risk cornea transplantation models have mainly involved the formation of a pre-vascularised graft bed, either by placing sutures into the recipient cornea 7–14 days prior to transplantation (4–6) or causing a chemical injury to the cornea prior to transplantation (7). These models result in ingress of blood and lymphatic vessels to the recipient graft bed which removes the immune privileged nature of the anterior chamber of the eye. This permits free access and egress of immune cells to the cornea, leading to accelerated graft rejection (4–7). Another high-risk transplant scenario arises when the allograft recipient has pre-existing anti-donor immunity—a situation that is likely to arise in the setting of re-transplantation following immune-mediated failure of one or more prior grafts. Indeed, a pre-existing immunity to a single antigen mismatch has been shown to significantly accelerate graft rejection in a mouse model (8).

Cornea transplantation has been recognized as a target for mesenchymal stromal cell (MSC) therapy for several years. A number of research groups, including our own, have demonstrated the therapeutic potential and mechanism of action of systemically-administered MSC in models of corneal allo-transplantation (9–14). It is widely accepted that MSC possess potent anti-inflammatory capabilities and modulate the activity of a range of immune effector cells including T cells, B cells, dendritic cells and macrophages (11, 14–16). This modulation occurs both through cell-cell contact and through the paracrine release of soluble factors such as nitric oxide (NO), prostaglandin E_2_ (PGE_2_), tumor necrosis factor-inducible gene 6 protein (TSG-6), indoleamine 2,3-dioxygenase (IDO) and several others (17–19). However, a number of critical questions remain to be answered in regard to the use of MSC in cornea transplantation; most notably whether autologous or allogeneic MSC represent the optimal cell source. In a conventional risk rat model, we have previously shown that autologous MSC are ineffective while allogeneic, either donor-derived or third-party, are highly effective at prolonging corneal allograft survival when administered prior to transplantation (11). In contrast, Omoto et al. showed, in a fully MHC-mismatched mouse model, that autologous MSC administered post transplantation significantly prolonging corneal transplant survival (12). MSC can be sourced from different tissues including bone marrow and adipose tissue. In the setting of corneal transplantation, tissue source of MSC may also have an impact on efficacy. A number of studies have demonstrated reduced rejection with administration of bone marrow-derived MSC (11, 12). In contrast, in a high-risk (pre-vascularized) rabbit model, Fuentes-Julien et al. reported lack of a beneficial effect of adipose-derived MSC injected intravenously at 4 separate time-points (20). Despite this result, the impact of dosing strategy, cell source, and allogenicity could all play significant roles, and further investigation of MSC efficacy in high-risk cornea transplantation is needed.

Another relevant concern with regard to the clinical translation of MSC immunomodulatory therapy for high-risk corneal transplantation is the co-administration of cells with immunosuppressive drugs. This issue has been studied to some extent in animal models of solid organ transplantation. For example, in a heart transplant model, Eggenhofer et al. showed that MSC and mycophenolate mofetil (MMF) had a synergistic effect when administered in combination which resulted in increased graft survival (21). Cell therapy approaches such as MSC also have other significant logistical challenges when being administered in a clinical setting. These include how the cells will be transported and stored and whether they require reconstitution at the bedside. For this reason, an “off the shelf” allogeneic cell product manufactured from healthy donors then delivered frozen and administered directly after thawing with no requirement for cell culture facilities at the clinical site is often considered to be optimal for the widespread clinical application of MSC and other cell therapies (22).

In this study, we aimed to determine whether intravenously delivered third-party MSC (fully allogeneic to both donor and recipient) were capable of prolonging rejection-free survival of corneal allografts in the setting of pre-existing anti-donor immunity. We also sought to investigate the potential *in vivo* immunomodulatory mechanisms of third-party allo-MSC in high-risk corneal transplant recipients and the feasibility of using a cryopreserved cell preparation in combination with the commonly prescribed immunosuppressant drug MMF.

## Materials and methods

### Cornea transplantation

Male Lewis (RT-1^l^) and Dark Agouti (DA; RT-1^avl^) rats aged 8–14 weeks were purchased from Envigo (Huntingdon, UK) and housed in a fully-accredited bio-resource. All procedures were approved by the NUI Galway Animal Care Research Ethics Committee and authorized by the Health Product Regulatory Authority (HPRA) of Ireland. Orthotopic corneal transplantation was performed on Lewis rats using DA donor corneas as reported previously (23). Corneal opacity was the primary indicator of graft rejection and was evaluated three times per week based on the following scale: 0-completely transparent cornea; 0.5-slight corneal opacity, iris structure easily visible; 1.0-low corneal opacity with visible iris details; 1.5-moderate corneal opacity, iris vessels still visible; 2.0-moderate opacity, only some iris details visible; 2.5-high corneal opacity, only pupil margin visible; 3.0-complete corneal opacity, anterior chamber not visible. Grafts were considered rejected if they reached an opacity score of ≥2.5 on two consecutive observations or a score of 3.0 on one occasion. Neo-vascularisation was assessed based on the number of quadrants of the donor cornea in which vessels were present. Corneal edema was quantified as central corneal thickness using a pachymeter (Micro Medical Devices, Calabasas, CA, USA) based on the following scale: 0-0-200 μm; 1-200-300 μm; 2-300-400 μm; 3-400 μm+. Animals with surgical complications were excluded.

### Pre-sensitisation

For donor-specific sensitization, splenocytes were isolated from healthy 6–12 weeks old male DA rats. Briefly, the spleen was isolated using aseptic technique post-mortem and stored in sterile phosphate buffered saline (PBS). Under a laminar flow hood, a single cell suspension was obtained by mashing the spleen through a 40 μm cell strainer (Fisher-Scientific, Wexford, Ireland). Red blood cells were lysed using ACK buffer for 5 min at room temperature. Splenocytes were washed and counted then re-suspended at a concentration of 20 × 10^6^ cells/ml in sterile PBS. Lewis rats were injected subcutaneously with 10 x 10^6^ DA splenocytes in 0.5 ml of sterile PBS 14 days prior to cornea transplantation.

### MSC culture, characterization, and administration

Wistar Furth (WF) rat MSC were isolated from the bone marrow of the femurs and tibiae of 6–10 week old male WF rats. Briefly, the rats were euthanised humanely and the bone of the legs dissected away under sterile conditions. The legs were transferred to a Biological Safety Cabinet and the bone marrow was flushed from the bones, red blood cells were lysed and the mononuclear cells were counted. Cells were seeded in tissue culture flasks at a density of 9 × 10^5^ cells per cm^2^ and cultured under standard culture conditions (24). MSC characterization was performed for standard surface markers by flow cytometry. (Supplementary Figure 1). For *in vivo* administration, MSC were trypsinised and counted then suspended at 1 × 10^6^ cells/ml in sterile PBS. For preparation of cryopreserved MSC, the cells were cultured to passage 2 (P2) then were lifted by trypsinization, washed, re-suspended at 1 × 10^6^ cells/ml in human serum albumin with 10% DMSO and cooled slowly to −80°C for 24 h before being transferred to liquid nitrogen. Aliquots of cryopreserved MSC were thawed rapidly in a 37°C water bath immediately prior to administration. Based on our previous data (11), freshly cultured or thawed, cryopreserved MSC were injected intra-venously through a tail vein at 7 days and 1 day prior to cornea transplantation. In the case of cultured MSC, cells were trypsinised and resuspended in PBS at a concentration of 1 × 10^6^ cells per ml. In the case of cryopreserved MSC, controlled animals were administered an equal volume of 10% DMSO in human serum albumin, while for cultured MSC treated animals, control animals received an equal volume of PBS. 1 × 10^6^ MSC were administered through the lateral tail vein using a 25 gauge needle slowly over 1 min.

### Mycophenolate mofetil preparation and administration

Mycophenolate mofetil (MMF; CellCept®, Roche Pharmaceuticals, Basel, Switzerland) was administered at a dose of 20 mg/kg daily from 1 day prior to 6 days post-transplant (Figure 7A) (21). MMF was resuspended in 5% glucose solution at a concentration of 20 mg/ml. Animals were weighed prior to injection and an appropriate volume of MMF was administered intra-peritoneally using an insulin syringe. Vehicle-treated animals were administered the corresponding volume of 5% glucose solution only.

### Allo-antibody assay

A total of 600 μl of blood was drawn from a tail vein at day 0 from pre-sensitized and naïve animals. The blood was layered onto Ficoll Paque (GE Healthcare Life Sciences, Buckinghamshire, UK) and centrifuged at 400 g for 20 min. Plasma was isolated by carefully removing the upper layer from the tube. Aliquots of 50 μl of plasma were incubated with 1 × 10^6^ donor derived (DA) splenocytes for 2 h at 4°C. The splenocytes were washed and incubated with FITC-conjugated anti-rat IgG2 antibodies for 40 min at 4°C. Mean fluorescence intensity was analyzed on a FACSCanto II (BD Biosciences, Franklin Lakes, NJ, USA) flow cytometer (25).

### Corneal histology and infiltrating cell quantification

Whole eyes were removed by dissection immediately after euthanasia. Eyes were fixed in 10% formalin and embedded in paraffin wax using an automatic tissue processor (Leica ASP300, Leica, Wetzlar, Germany). Sections of 5 μm thickness were cut on a rotary microtome (Leica) and fixed to glass slides. Hematoxylin and eosin staining was performed as previously described (11). Sections were imaged at 10X magnification and images taken using an Olympus BX61 microscope (Olympus, Tokyo, Japan). Infiltrating cells were quantified by counting the number of distinct cells visible in the corneal stroma of 3 separate 10X fields of view per animal.

### Immune cell profiling

Animals were euthanized at day 0 and day 10 for immune cell profiling experiments. Spleen, lungs and draining lymph nodes were dissected immediately after euthanasia. Single cell suspensions were prepared from spleens and lymph nodes by mashing the tissue through a 40 μm cell strainer with PBS. Single cell suspensions were obtained from lungs by first cutting the lungs into small (~2 mm^2^) pieces and digesting for 2 h at 37°C in 100 U/μl Collagenase IV (Sigma-Aldrich) and 200 U/μl DNase I (Bioline, London, UK) followed by mashing through a 40 μm cell strainer. Single cell suspensions were stained with fluorochrome-conjugated antibodies and data were acquired on a FACSCanto II (BD Biosciences) flow cytometer. Data were analyzed using FlowJo flow cytometry analysis software (Tree Star Inc., Ashland, OR, USA).

### Co-culture experiments

CD11b/c^+^ myeloid cells were enriched from lung-derived cell suspensions by magnetic column separation using anti-rat CD11b/c microbeads (Miltenyi Biotec, Bergisch Gladbach, Germany) according to the manufacturer's recommended protocol. Isolated CD11b/c^+^ cells were co-cultured at a ratio of 1:1 with MSC in a 96-well round-bottomed plate for 48 h in a humidified incubator at 37°C and 5% CO_2_. Supernatants were saved and CD11b/c^+^ cells were re-enriched from the mixture by the same magnetic column separation procedure.

Whole lymph nodes cells were prepared from the subcutaneous lymph nodes of healthy Lewis rats between 5 and 12 weeks of age. The lymph nodes cells were washed with PBS and stained with CellTrace Violet (Fisher Scientific Ltd., Dublin, Ireland) according to the manufacturer's recommended protocol. Aliquots of 1 x 10^5^ CellTrace Violet-labeled lymph node cells were added to the wells of 96-well round-bottomed tissue culture plates with or without anti-rat CD3/CD28 beads at 1:1 ratio in T cell medium (RPMI 1,640 supplemented with 10% fetal calf serum, 50 μM β-mercaptoethanol, 100 U/ml penicillin, 0.1 mg/ml streptomycin, 1 mmol/l sodium pyruvate and 2 mmol/l L-Glutamine). In various experiments, MSC were added at T cell:MSC ratio of 50:1 or CD11b/c^+^ cells were added at 2:1 T cell:myeloid cell ratio for 4 days prior to analysis by flow cytometry.

For flow cytometry analysis, T cells were stained with fluorochrome conjugated antibodies for surface expression of CD4, CD8, and CD25. Intracellular staining for FoxP3 was performed according to the recommended protocol using the FoxP3 staining buffer kit (Fisher Scientific Ltd.). Briefly, cells were first stained for surface expression of CD4, CD8, and CD25 and washed in FACS buffer (PBS, 2% FBS and 0.05% Sodium Azide). Cells were then resuspended in 200 μl Fixation/Permeabilisation buffer and incubated at 4°C overnight. The next morning, the cells were washed twice with permabilisation buffer and stained with anti-FoxP3-PE-Cy7 (eBioscience) in permeabilisation buffer for 45 min at 4°C. Finally, cells were washed once more in permeabilisation buffer, re-suspended in FACS buffer and analyzed for surface marker expression, proliferation and FoxP3 expression on a FACSCanto II flow cytometer. Proliferation was analyzed using FlowJo software by identifying each generation on the histogram and comparing the proliferation of MSC or myeloid-containing wells with stimulated or unstimulated lymph node cells.

### Assays for nitric oxide (NO), prostaglandin E_2_ (PGE_2_), and transforming growth factor β (TGFβ)

PGE_2_ and TGFβ1 ELISA kits were purchased from R&D Systems, Abingdon, UK. Analyte levels were detected using these kits according to manufacturer's instructions. The presence of nitric oxide (NO) in culture supernatants was analyzed by Griess assay. Aliquots of 100 μl of supernatant were added to 96-well flat-bottom optical plates and an equal volume of Griess reagent (1% sulphanilamide and 0.1% N-1-(naphthyl)-ethylenediamine-diHCl in 2.5% H_3_PO_4_) was added to each well. Absorbance was measured immediately at 540 nm on a plate reader (Perkin Elmer, Dublin, Ireland). NO concentration was calculated using a sodium nitrite standard curve (24).

### Statistical analysis

All data were analyzed using GraphPad Prism software, version 6 (GraphPad Software, La Jolla, CA, USA) and were expressed as mean ± SEM. Log-rank (Mantel-Cox) or one-way ANOVAs with Tukey's post-test were used to determine statistical differences among groups as appropriate. For all experiments, statistical significance was assigned at *p* < 0.05.

## Results

### Generation of pre-sensitized cornea transplant model

**Figure 1:**
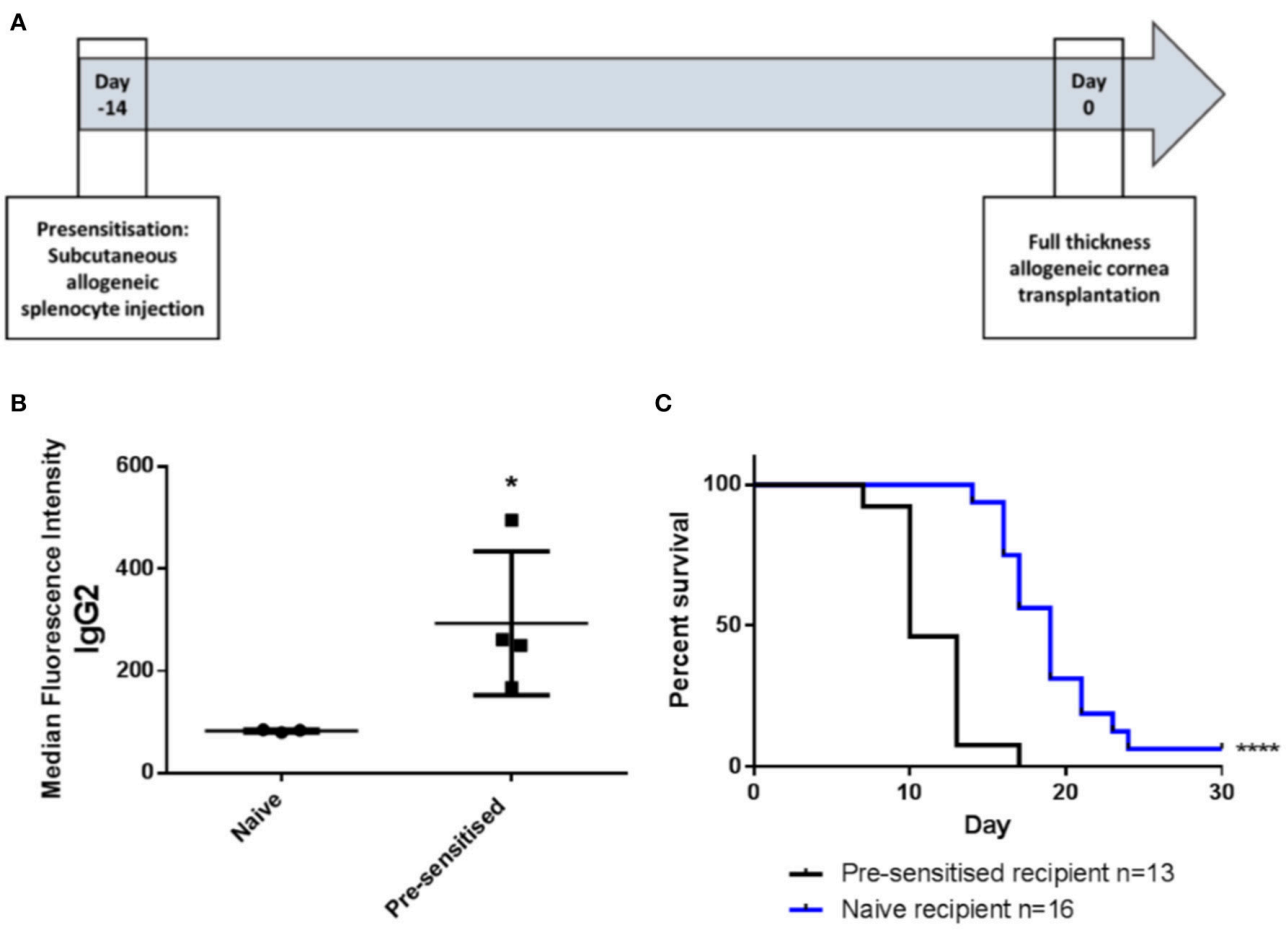
DA-splenocyte pre-sensitized Lewis rats develop anti-DA IgG antibodies and reject corneal allografts earlier than non-pre-sensitized Lewis rats. **(A)** Lewis rats were pre-sensitized with 10 × 10^6^ DA rat (donor) splenocytes 14 days prior to cornea transplantation. **(B)** Graph showing results of flow cytometry analysis of DA rat splenocytes incubated with serum samples taken at the time of transplantation from non-pre-sensitized (naïve; *n* = 3) and pre-sensitized (*n* = 4) Lewis rats followed by staining with anti-rat-IgG2-FITC. Individual results (symbols) and mean ± SEM for the two groups are shown and expressed as median fluorescence intensity on the FL1 channel. **(C)** Kaplan-Meier plots depicting DA to Lewis rat corneal allograft rejection-free survival in naïve (*n* = 16; blue line) and pre-sensitized (*n* = 13; black line) animals. Statistical significance was determined using a one tailed Student's *t*-test **(B)** or Log-rank (Mantel-Cox) test **(C)** as appropriate ^*^p < 0.05, ^****^p < 0.0001.

Pre-sensitization of Lewis rats to DA allo-antigens was confirmed by detection of anti-DA IgG2 antibodies in serum samples from rats that had received subcutaneous inocula of DA splenocytes 14 days previously but not in sera from naïve Lewis rats (Figures 1A,B) (11, 24). Subsequently, in DA-to-Lewis corneal allotransplants, pre-sensitized graft recipients were shown to have accelerated rate of rejection compared to naïve recipients (Figure 1C). In this experiment, average day of rejection (ADR) for pre-sensitized recipients was 11.46 compared to 19.25 for naïve recipients. It was concluded that the inoculation of recipients with corneal donor strain splenocytes 14 days prior to transplantation provided a robust, high immunological risk model in which to investigate the effects of third-party allo-MSC administration.

### Intravenous administration of third-party allo-MSC increases rejection-free survival of high immunological risk corneal transplants

**Figure 2:**
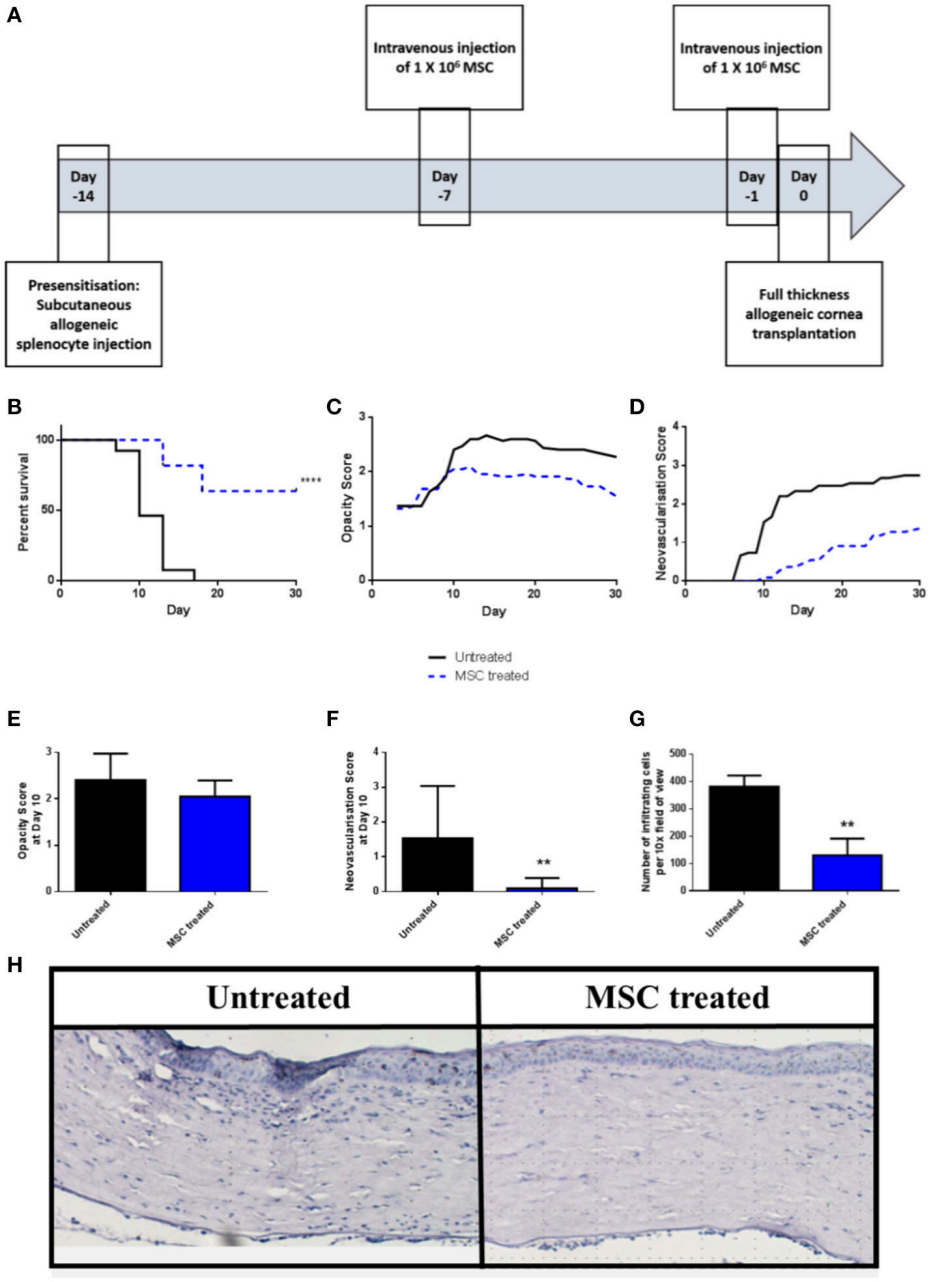
Third-party allo-MSC are capable of prolonging rejection-free corneal allograft survival in pre-sensitized recipients. **(A)** Schematic representation of administration of third-party allo-MSC in the pre-sensitized high-risk rat corneal transplant model. **(B)** Kaplan-Meier curves demonstrating rejection-free survival of pre-sensitized Lewis recipients of DA corneas that received third-party allo-MSC injections (MSC-treated, *n* = 13; blue dashed line) or vehicle injections (Untreated, *n* = 11; black line) up to 30 days post-transplant. **(C,D)** Trend-lines for corneal allograft opacity scores **(C)** and neovascularisation scores of the two groups of pre-sensitized corneal transplant recipients up to day 30 post-transplant. **(E–G)** Corneal allograft opacity scores **(E)**, neovascularisation scores **(F)** and number of corneal infiltrating cells per 10X field **(G)** on day 10 post-transplant for untreated (black bar) and MSC treated (blue bar) groups. All data are presented as mean ± SEM. **(H)** Representative examples of H&E stained sections of corneal allografts of untreated and MSC-treated pre-sensitized Lewis rats at day 10 post-transplant. Statistical significance was determined using a Log-rank (Mantel-Cox) test or one-tailed Student's *t*-test or as appropriate ^**^p < 0.01, ^****^p < 0.0001.

We have previously shown that a protocol consisting of two pre-transplant intravenous injections of third-party allo-MSC is similarly efficient to corneal donor-specific allo-MSC at modulating graft rejection in a conventional risk rat corneal transplant model (11). Using the same injection strategy (Figure 2A), it was observed that the rejection rate of corneal transplants in pre-sensitized recipients was strongly reduced by intravenous injections of third-party allo-MSC at day−7 and day−1 (Figure 2B). In this experiment, the 30-day rejection-free survival of MSC-treated animals was 63.6% compared to 0% in the untreated group. In keeping with a potent immunomodulatory effect, the trends for mean graft opacity and neovascularisation scores were lower for MSC-treated animals throughout the 30-day post-transplant observation period (Figures 2C,D). At day 10, which approximates the ADR for untreated animals in this model, the mean opacity score of MSC-treated animals was 2.05 ± 0.11 compared to 2.40 ± 0.15 for the controls (Figure 2E). At this same, key time-point, mean neovascularisation scores for the MSC- and control-treated animals were 0.09 ± 0.09 and 1.53 ± 0.39 (Figure 2F) and the mean numbers of infiltrating cells, as determined by analysis of H&E stained tissue sections, were 130.33 ± 61.72 and 382.33 ± 39.7 respectively (Figures 2G,H). Thus, in the pre-sensitized, high immunological risk rat corneal allograft model, administration of third-party allo-MSC exerted a potent protective effect against acute rejection that was comparable to the previously-reported effect in a conventional risk model.

### MSC administration results in localized, proportionate increases in regulatory immune cells

**Figure 3:**
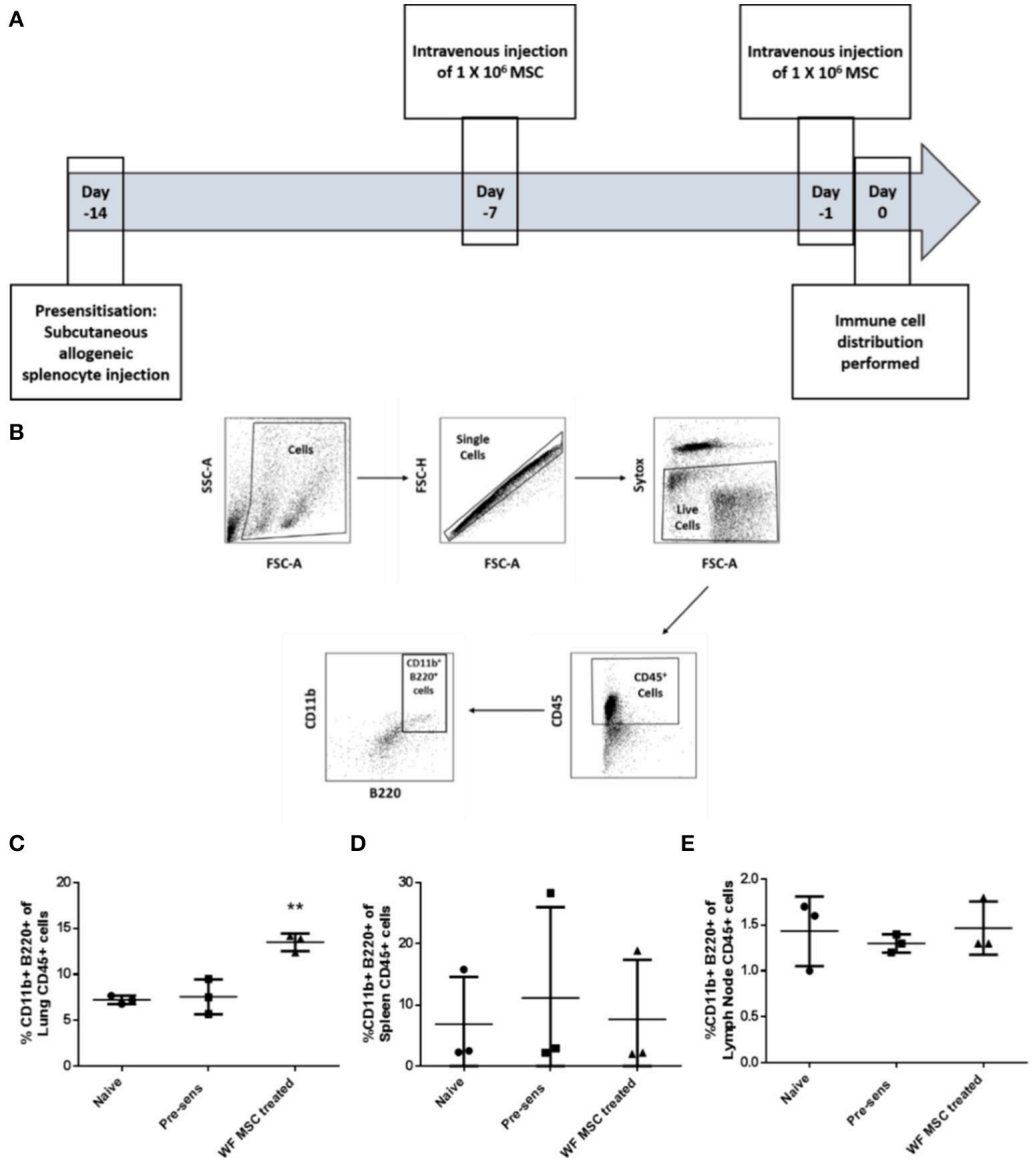
Third-party allo-MSC administration to pre-sensitized Lewis rats results in a proportionate increase in lung CD11b^+^/B220^+^ cells on the day of transplantation. **(A)** Schematic representation of experiment to evaluate immune cell profiles in pre-sensitized Lewis rats treated with third-party allo-MSC 1 and 7 days earlier**. (B)** Gating strategy used to gate CD11b^+^ B220^+^ cells from tissues of transplanted rats **(C–E)** Graphs showing results of flow cytometry analysis of the proportion of CD45^+^ cells co-expressing CD11b and B220 in the lungs **(C)**, spleens **(D)** and submandibular lymph nodes **(E)** of non-pre-sensitized/untreated rats (naïve, *n* = 3); pre-sensitized/untreated rats (Pre-sens, *n* = 3) and pre-sensitized/third-party allo-MSC treated rats (WF MSC treated, *n* = 3). Results for individual animals (symbols) and mean ± SEM for each group (horizontal lines with error bars) are shown. Statistical analysis was performed using one-way ANOVA with Tukey's post-test, ^**^p < 0.01.

In order to study the effects of third-party allo-MSC therapy on the dynamics of potential regulatory immune cell populations, samples of various organs from three groups of Lewis rats (naïve, pre-sensitized untreated and pre-sensitized allo-MSC-treated) were analyzed at two time-points by multi-color flow cytometry. On the prospective day of transplantation (day 0), cell suspensions from lungs, spleens and draining (submandibular) lymph nodes were assessed for immune cell subtypes by multi-color flow cytometry (Figures 3A,B). In pre-sensitized, allo-MSC-treated animals, higher mean proportions of lung CD45^+^ cells were double-positive for CD11b and B220 (13.5%) in comparison to naïve (7.2%) and untreated pre-sensitized (7.6%) animals (Figure 3C). No differences in the proportions of CD11b^+^ B220^+^ cells were observed among the total CD45^+^ populations of the spleens and draining lymph nodes from the 3 groups at this time-point (Figures 3D,E). Of note, CD45^+^ CD11b^+^ B220^+^ cells have been shown to be increased in the lungs of mice following administration of human MSC and to be sufficient for prolongation corneal allograft survival (13). No significant changes in proportions of T cell populations were observed in any of the organs examined at this time-point (data not shown). It should be noted that MSC have also previously been reported to migrate to other tissues, including spleen, lymph nodes, thymus and others and that these cells could exert effects in cells in these organs or in the circulation which were not detected here.

**Figure 4:**
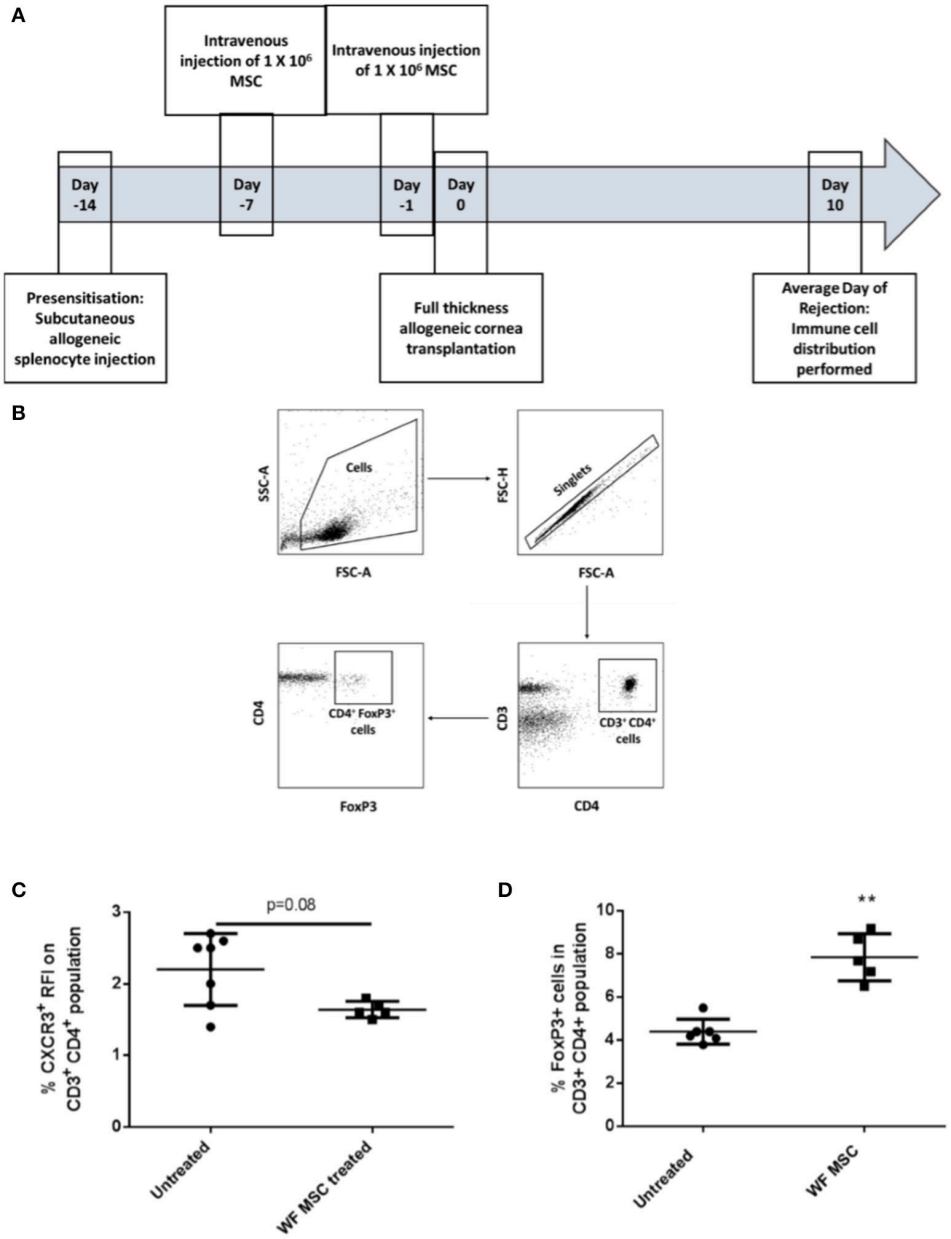
Third-party allo-MSC administration to pre-sensitized Lewis rat corneal transplant recipients results in a proportionate increase in draining lymph node FoxP3^+^ (regulatory) CD4^+^ T-cells cells 10 days after transplantation. **(A)** Schematic representation of experiment to evaluate immune cell profiles at day 10 post-transplant in pre-sensitized Lewis rat recipients of DA corneal allografts following treatment with third-party allo-MSC. **(B)** Gating strategy used to identify FoxP3^+^ cells by flow cytometry (C and D). Graphs showing results of flow cytometry analysis of the proportions of CD3^+^CD4^+^ T-cells co-expressing CXCR3 **(C)** and FoxP3 **(D)** in the graft-draining lymph nodes of pre-sensitized/untreated rats (Pre-sens, *n* = 6) and pre-sensitized/third-party allo-MSC treated rats (WF MSC treated, *n* = 5). Results for individual animals (symbols) and mean ± SEM for each group (horizontal lines with error bars) are shown. Statistical analysis was performed using one-way ANOVA with Tukey's post-test, ^**^p < 0.01.

The same immunological profiling was then carried out on tissues from groups of pre-sensitized animals that had been transplanted 10 days previously and treated either with third-party allo-MSC or untreated (Figure 4A). At this time-point, a trend toward a reduction in the proportion of CD3^+^ CD4^+^ CXCR3^+^ Th1 cells in the draining lymph node was observed (Figure 4C) as well as an increase in the proportion of CD3^+^ CD4^+^ FoxP3^+^ regulatory T cells (mean 4.4% in untreated compared to 7.9% in MSC-treated, pre-sensitized animals; Figures 4B,D). Importantly, increased regulatory T cells has been previously shown to prolong corneal allograft survival (5). These changes in proportions of T cell populations were only observed in the draining lymph nodes, indicating a localized corneal transplant-specific effect.

### MSC-educated lung myeloid cells exhibit an immunosuppressive phenotype

**Figure 5:**
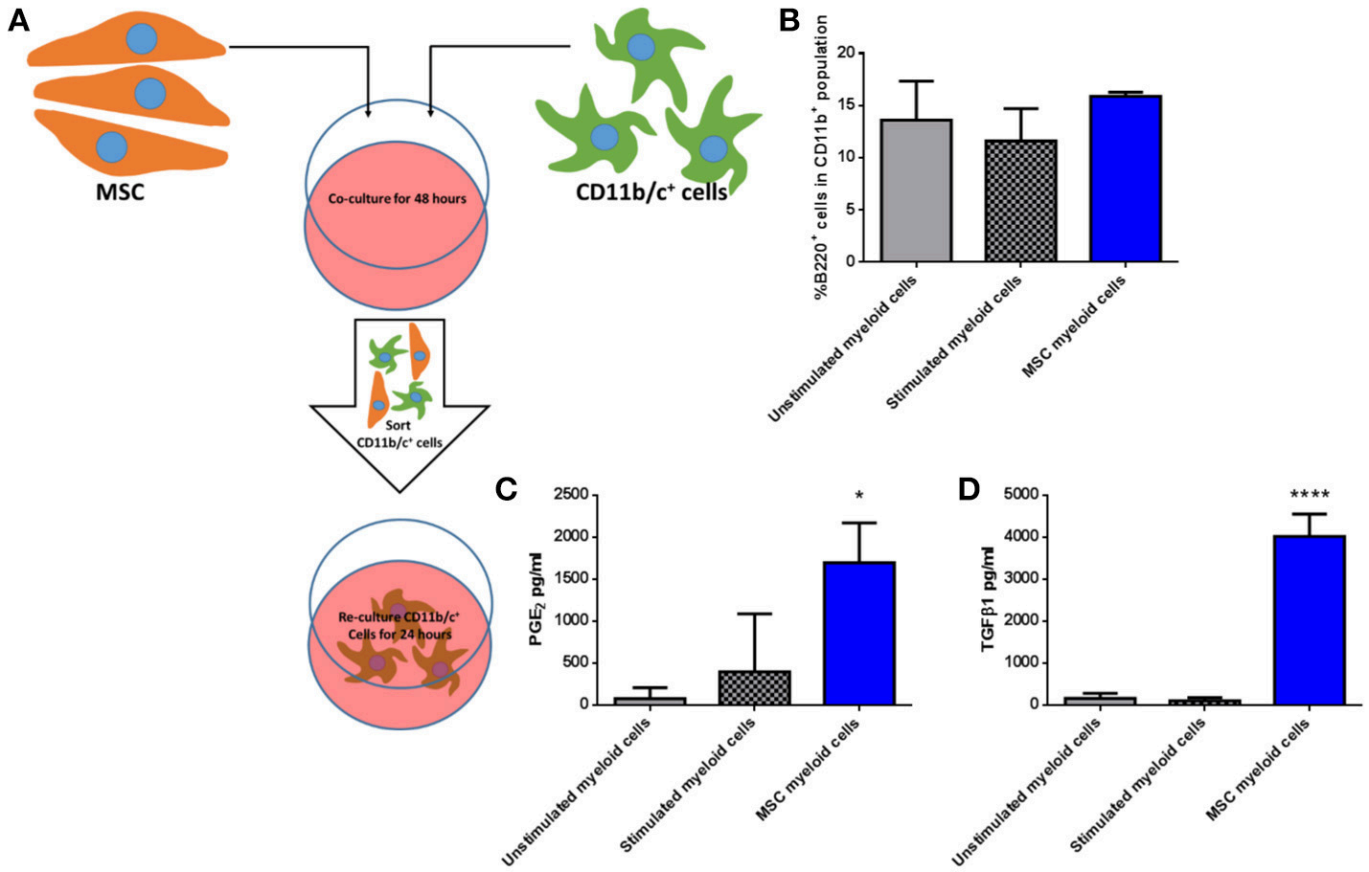
Allogeneic MSC polarize macrophages to a more immunomodulatory phenotype *in vitro* **(A)** Schematic representation of the co-culture of CD11b/c^+^ (myeloid) cells isolated from Lewis rat lungs with Wistar Furth MSC or control conditions for 48 h followed by re-purification and culture for 24 h. **(B)** Graph of the proportions of B220^+^ cells among CD11b^+^ cells following 48 h culture Lewis rat lung myeloid without further stimulus (Unstimulated myeloid cells), with IFNγ/LPS stimulation (Stimulated myeloid cells) and with Wistar Furth MSC (MSC myeloid cells). **(C,D)** Graphs of the concentrations of PGE_2_ **(C)** and TGFβ1 **(D)** in 24 h culture supernatants of CD11b/c^+^ Lewis rat lung myeloid cells re-purified from the same three 48-h culture conditions. All results are presented as mean ±SEM with *n* = 3 in each group. Statistical analyses were performed using one way ANOVA with Tukey's post-test, ^*^p < 0.05, ^****^p < 0.0001.

To determine if third party allo-MSC directly increase the proportion of potentially regulatory CD45^+^CD11b^+^ B220^+^ cells among native lung myeloid cells *ex vivo*, CD11b/c^+^ cells were isolated from the lungs of naïve Lewis rats by magnetic column separation. The isolated cells were then cultured for 48 h under 3 conditions: Unstimulated, stimulated with IFNγ/LPS alone and cultured in the presence of WF allo-MSC for 48 h (Figure 5A). CD11b/c^+^ cells were then re-separated from MSC by magnetic column separation and cultured alone for 24 h. In this experiment, however, no increase in the proportion of CD45^+^CD11b^+^B220^+^ cells was observed following allo-MSC co-culture, suggesting that the higher proportion of B220^+^ myeloid cells in lungs of allo-MSC-treated animals was not explained by direct induction of this marker on resident, CD11b^+^B220^−^ myeloid cells (Figure 5B). Nonetheless, significantly higher concentrations of PGE_2_ and TGFβ1 were generated by myeloid cells that had been re-purified from cultures containing allo-MSC compared to unstimulated or IFNγ/LPS stimulated myeloid cells (Figures 5C,D).

**Figure 6:**
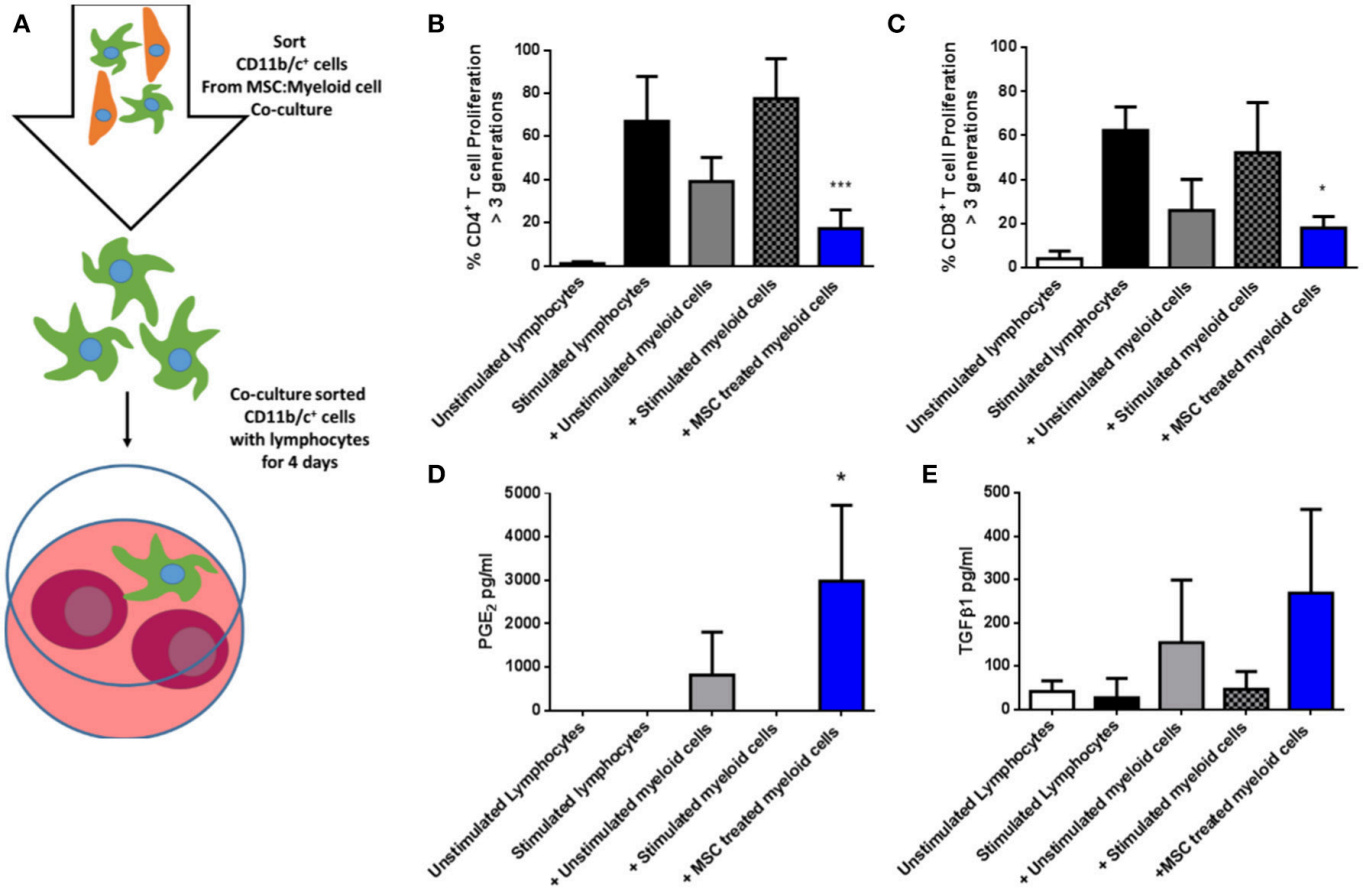
Allogeneic MSC-educated lung myeloid cells more potently suppress T-cell proliferation. **(A)** Schematic representation of the addition of Lewis rat lung CD11b/c^+^ (myeloid) cells re-sorted from co-cultures with Wistar Furth MSC or control conditions and then added to syngeneic lymphocytes, with, or without anti-CD3/anti-CD28 stimulation for 4 days. **(B,C)** Graphs of CD4^+^ **(B)** and CD8^+^ **(C)** T-cell proliferation, quantified by flow cytometry-based fluorescent dye dilution as the proportion of cells that had proliferated through >3 generations, in 4-day cultures of Lewis rat splenocytes under five conditions: No stimulation (Unstimulated lymphocytes); Anti-CD3/CD28 stimulation alone (Stimulated lymphocytes); Anti-CD3/CD28 stimulation + re-purified unstimulated Lewis rat lung myeloid cells + Unstimulated myeloid cells; Anti-CD3/CD28 stimulation + re-purified, IFNγ/LPS-stimulated Lewis rat lung myeloid cells (+ Stimulated myeloid cells) and Anti-CD3/CD28 stimulation + re-purified Wistar Furth MSC co-cultured Lewis rat lung myeloid cells (+ MSC treated myeloid cells). **(D,E)** Graphs of the concentrations of PGE_2_ **(D)** and TGFβ1 **(E)** in 4-day Lewis rat splenocyte cultures under the same five conditions. All data are presented as mean ± SEM with *n* = 3 in each condition. Statistical analysis was performed using one way ANOVA with Tukey's post-test, ^*^p < 0.05, ^***^p < 0.001.

Next, CD11b/c^+^ cells re-isolated from the three culture conditions were co-cultured with syngeneic fluorescently-labeled lymphocytes in the presence of anti-CD3/anti-CD28 stimulation for 4 days (Figure 6A), following which proliferation was quantified by flow cytometry based on dilution of the fluorescent dye. As shown in Figures 6B,C, MSC-educated lung myeloid cells suppressed the proliferation of CD4^+^ and CD8^+^ T cells to a greater degree that unstimulated lung myeloid cells, while IFNγ/LPS stimulated lung myeloid cells had no suppressive effect. This suppression was accompanied by an increase in the PGE_2_ concentration (Figure 6D) as well as a trend toward an increase in TGFβ1 (Figure 6E) in the culture supernatants of the T cell stimulation cultures containing MSC-educated lung myeloid cells. It was concluded that direct contact between allo-MSC and primary lung myeloid cells results in a deviation of the myeloid cell activation response toward an anti-inflammatory, immunosuppressive phenotype.

### Cryopreserved MSC are capable of prolonging corneal allograft survival

**Figure 7:**
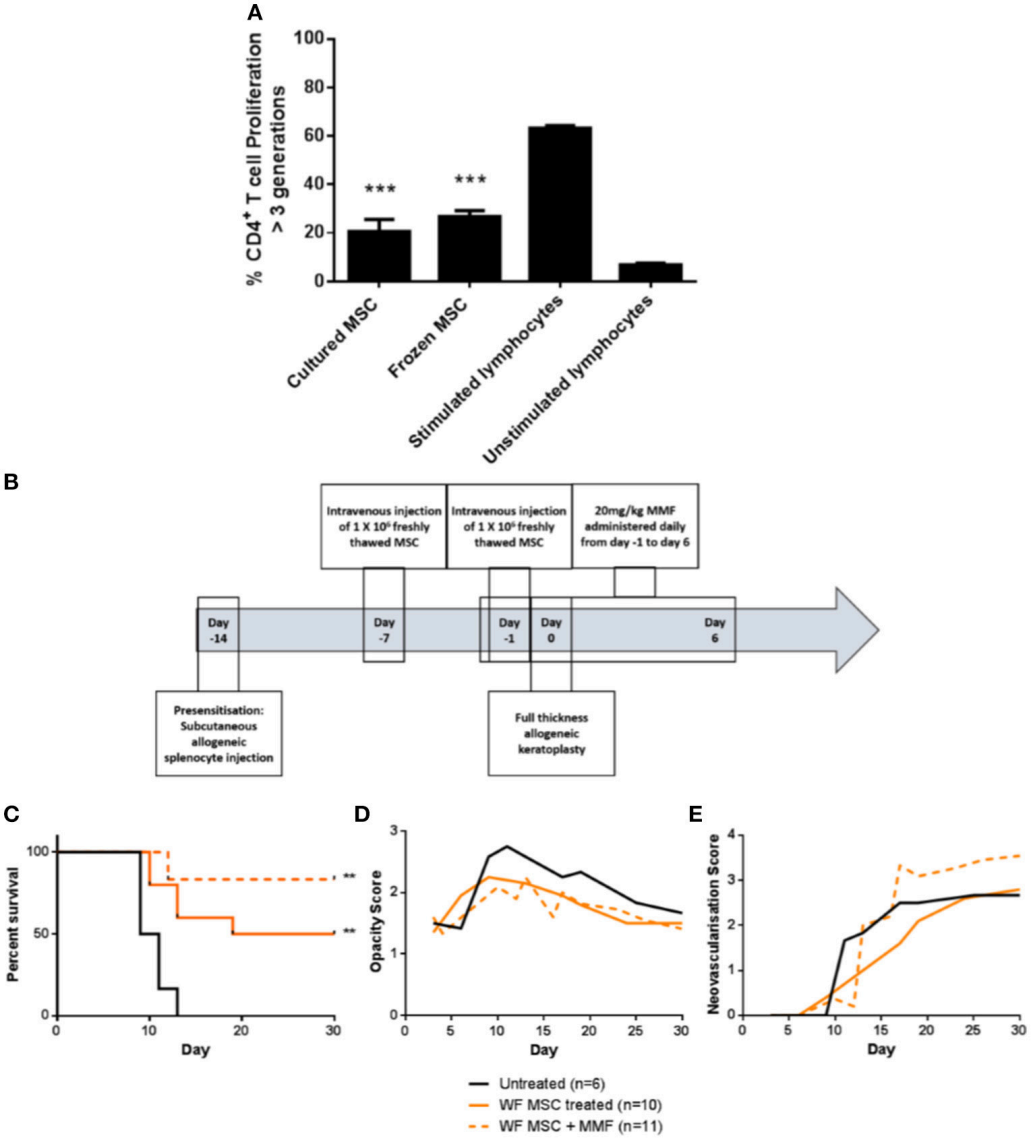
Cryopreserved third party allo-MSC are immunosuppressive and capable of prolonging corneal allograft survival without and with mycophenolate mofetil co-administration in pre-sensitized recipients. **(A)** Graph of CD4^+^ T-cell proliferation, quantified by flow cytometry-based fluorescent dye dilution as the proportion of cells that had proliferated through >3 generations, in 4-day cultures of Lewis rat splenocytes under four conditions: Anti-CD3/CD28 stimulation + freshly cultured Wistar Furth MSC (Cultured MSC); Anti-CD3/CD28 stimulation + thawed, cryopreserved Wistar Furth MSC (Frozen MSC); Anti-CD3/CD28 stimulation alone (Stimulated lymphocytes) and No stimulation (Unstimulated lymphocytes). **(B)** Schematic representation of administration of thawed, cryopreserved (freshly thawed) third-party allo-MSC and MMF in the pre-sensitized high-risk rat corneal transplant model. **(C)** Kaplan-Meier curves demonstrating rejection-free survival of pre-sensitized Lewis recipients of DA corneas that received no treatment (Untreated, *n* = 6); thawed, cryopreserved third-party allo-MSC (WF MSC, *n* = 10) and thawed, cryopreserved third-party allo-MSC with MMF (WF MSC + MMF, *n* = 11). **(D,E)** Trend-lines for corneal allograft opacity scores **(D)** and neovascularisation scores **(E)** of the same three groups of pre-sensitized corneal transplant recipients up to day 30 post-transplant. Statistical significance was determined using a Log-rank (Mantel-Cox) test, ^**^p < 0.01 compared to Untreated group. ^***^p < 0.001.

For translation of an allogeneic cell therapy to the clinic, logistical concerns like transport of cells from the production site to the clinical site, formulation of the sample and manipulations required at the bedside become major concerns (26). For instance, the ability to thaw and directly administer cells at the bedside may be critical for clinical cost-effectiveness and ease of use. To address this issue in the context of high-risk corneal transplantation, *in vitro* and *in vivo* experiments were carried out using freshly-thawed cryopreserved third-party allo-MSC. As shown in Figure 7A, thawed, cryopreserved allo-MSC mediated equivalent suppression of CD4^+^ T cell proliferation as freshly cultured cells. Furthermore, when administered to pre-sensitized animals at the same dose and time-points as for experiments using freshly cultured cells (Figure 7B), thawed, cryopreserved, third-party allo-MSC proved similarly capable of modulating corneal transplant rejection (Figures 7C–E). Cryopreserved allo-MSC-treated animals had an ADR of 21.5 days compared to 10.3 days for vehicle-treated animals.

Finally, to determine whether the allo-MSC-associated immunomodulation is compatible with peri-transplant administration of a clinically-relevant immunosuppressant, a group of pre-sensitized DA-to-Lewis corneal transplant recipients was treated with a combination of thawed, cryopreserved third allo-MSC and daily injections of MMF between day−1 and day 6 post-transplant (Figure 7B). As shown in Figure 7C, the combined treatment also resulted in a significant prolongation of corneal allograft rejection-free survival with ADR of 27. It was concluded that, in addition to their potent and consistent immunomodulatory effects, the translational potential of allo-MSC for amelioration of rejection in high immunological risk cornea transplant recipients is further enhanced by their compatibility with cryopreservation and with co-administration with MMF.

## Discussion

Patients at higher risk of corneal allograft rejection due to pre-existing inflammation or anti-donor immunity suffer from immune-mediated corneal rejection at an earlier time and higher frequency than patients classified as non-high-risk (1–3). A major issue in the clinical management of high-risk corneal allograft recipients is the reluctance to administer systemic immunosuppressive drug treatments due to the risk of side effects and the fact that, unlike other organ transplants, a corneal transplant failure is not a life threatening event (27, 28). This means that high-risk patients present a potential unmet clinical need which could be ameliorated with MSC therapy.

Pre-sensitization with donor-derived splenocytes resulted in an earlier and very consistent rejection profile in the DA-to-Lewis rat cornea transplantation model. This rejection was associated with rapid opacification and neovascularisation of the graft and a demonstrable pre-existing allo-immunity in the form of anti-DA IgG2 antibodies. Similar to our previous results in a conventional risk rat corneal transplant model, we show here that third-party allo-MSC are capable of significantly prolonging corneal allograft survival to day 30 in this high-risk model (11). Third-party MSC are the most relevant cells to use as, in the clinical setting for MSC therapies, it is likely that cells from a source allogeneic to both donor and recipient will have to be used for reasons of logistics and cost (11). Additionally, reports in recent years have shown the importance of activation of MSC with pro-inflammatory cytokines such as interferon-γ, tumor necrosis factor-α and interleukin-1β for their ability to manifest immunomodulatory effects (29–31). *In vivo*, this stimulus can come from introducing the MSC to an already-inflamed environment or through allo-recognition of the cells by the host immune system with subsequent production of pro-inflammatory factors (25, 32, 33). Thus, infusing an allogeneic cell, such as the Wistar Furth MSC employed in this study, may result in allo-recognition upon infusion and subsequent activation of the MSC by the host's immune system (18, 19).

The first organ which intravenously-infused MSC encounter is the lung. It is well established in animal models that MSC become lodged in the lungs soon after infusion and that the vast majority do not migrate to other tissues (10, 13, 34). In addition to residing in the lung after infusion, the half-life of infused MSC is remarkably short considering the long-term benefits they can exert (10, 13, 34). Ko et al. in an important recent study, showed that intravenously administered MSC primed CD11b^+^ B220^+^ lung myeloid cells in the mouse to prevent corneal allograft rejection (13). Here we show that these cells are also increased in the lungs of pre-sensitized high-risk rat cornea transplant recipients 24 h after administration of the second of two third-party allo-MSC infusions. The data presented here add to previous studies showing that MSC are transient mediators which may imprint their immunomodulatory phenotype onto relevant recipient cells to indirectly exert their long-term effect. In the case of this study, evidence of imprinting of MSC immunomodulatory phenotype included an almost 2-fold increase in the proportion of regulatory T cells in the draining lymph node during the time-period in which rejection typically manifests in this high-risk model. Regulatory T cells in the draining lymph node have been shown to be critically important for corneal allograft survival (35, 36) and depletion of these cells negatively impacts the survival of the graft (37). Indeed, several groups have shown that adoptive transfer of regulatory T cells and other *in vivo* strategies to increase the numbers of regulatory T cells can have beneficial effects on transplant survival (5, 38–40).

In *in vitro* experiments, we examined whether third-party allo-MSC directly induced an increase in the proportion of CD11b^+^ B220^+^ within a purified population of CD11b/c^+^ lung cells. However, we observed that there was no increase in the proportion of these cells in a co-culture with CD11b/c^+^ lung cells and MSC suggesting that, once localized to the lung, MSC may recruit CD11b^+^ B220^+^ and polarize these cells to a more immunomodulatory phenotype rather than switching the expression of previously lung resident CD11b^+^ B220^−^ cells. The production of immunomodulatory proteins such as TGF-β1 and PGE_2_ could polarize these CD11b/c^+^ cells to a more immunomodulatory phenotype. Third-party allo-MSC-educated CD11b/c^+^ cells were furthermore demonstrated to have enhanced anti-proliferative potency against activated T cells and to release immunomodulatory mediators such as PGE_2_ which, in addition to the above mentioned reduction in pro-inflammatory mediators, could all combine to result in an increase in the proportion of regulatory T cells and subsequent promotion of allograft survival.

Translation of a cell therapy such as MSC will likely involve one site producing cells for transport to and administration at several clinical sites. A therapy which can be stored, easily transported and administered in the clinic without a requirement for cell culture facilities is, therefore, ideal to benefit the maximum number of patients. A frozen cell product which can be thawed and directly administered to the patient fulfills the requirements for an easily adoptable cell therapy. Therefore, we determined whether MSC that had been cryopreserved in HSA/DMSO could prolong corneal allograft survival in the pre-sensitized model. Despite existing evidence that cryopreserved MSC may have impaired immunomodulatory capacities, including reduced expression of immunosuppressive molecules and impaired responses to pro-inflammatory stimuli (41), we found that cryopreserved MSC were equally capable of prolonging allograft survival as cultured cells. This important finding further demonstrates the translatability of an MSC therapy for high-risk cornea transplant patients who may have pre-existing allo-immunity.

Additionally, in a high-risk cornea transplant setting, it is likely that clinicians will co-administer MSC with an already established immunosuppressive drug regimen. We sought, therefore, to determine whether a peri-transplant course of MMF would augment or interfere with the efficacy of MSC in the high-risk pre-sensitized CT model. MMF was chosen for several reasons. Firstly, unlike other immunosuppressive drugs such as corticosteroids, it does not alter the expression of MSC immunomodulatory factors (42) and has been shown to exert a synergistic effect with MSC in a pre-clinical heart transplant model (21). Secondly, MMF is currently prescribed to patients receiving high-risk cornea transplants including re-grafts (43, 44). Our observation that co-administration of MMF did not diminish the anti-rejection of third-party allo-MSC therapy (and trended toward further increase in rejection-free survival) in this high-risk model provides additional evidence of clinical translational potential.

In conclusion, we have shown for the first time that pre-transplant intravenous administration of third-party allo-MSC results in distinct immune modulatory effects that overcome pre-existing anti-donor immunity to prolong rejection-free survival of corneal allografts in a rat model. Furthermore, the experimental cell therapy regimen described here appears to be compatible with prior cryo-preservation and with co-administration of a relevant immunosuppressive drug. These findings open the door to clinical translation of off-the-shelf allo-MSC products for recipients of high-risk corneal transplants who continue to suffer from very poor long-term graft survival rates. The results of this study, particularly those related to the co-administration of cryo-preserved allo-MSC and MMF have formed the basis of a regulatory submission for a Phase Ib clinical trial in corneal re-graft patients. This trial will determine the safety and feasibility of co-administration of allo-MSC and MMF in the setting of high risk cornea transplantation.

## Author contributions

When determining authorship, the following criteria should be observed: Substantial contributions to the conception or design of the work PL, MG, and TR or the acquisition PL, NM, OT, KL, MM, and BC analysis PL, NM, OT, KL, MM, and BC or interpretation of data for the work PL, AR, MG, and TR. Drafting the work or revising it critically for important intellectual content PL, AR, MG, and TR. Final approval of the version to be published PL, NM, OT, KL, MM, BC, AR, MG, and TR. Agreement to be accountable for all aspects of the work in ensuring that questions related to the accuracy or integrity of any part of the work are appropriately investigated and resolved PL, NM, OT, KL, MM, BC, AR, MG, and TR.

### Conflict of interest statement

The authors declare that the research was conducted in the absence of any commercial or financial relationships that could be construed as a potential conflict of interest.

## Supplementary material

Supplementary Figure 1 MSC were characterized by flow cytometry using standard markers **(A)** CD90, **(B)** CD73, **(C)** MHC-I, **(D)** MHC-II, **(E)** CD45, and **(F)** CD86.
