# Supplementary figures and images for: Third-Party Allogeneic Mesenchymal Stromal Cells Prevent Rejection in a Pre-sensitized High-Risk Model of Corneal Transplantation
Source: Front Immunol. 2018 Nov 20;9:2666. doi: 10.3389/fimmu.2018.02666 (PMC6255848; doi:10.3389/fimmu.2018.02666)

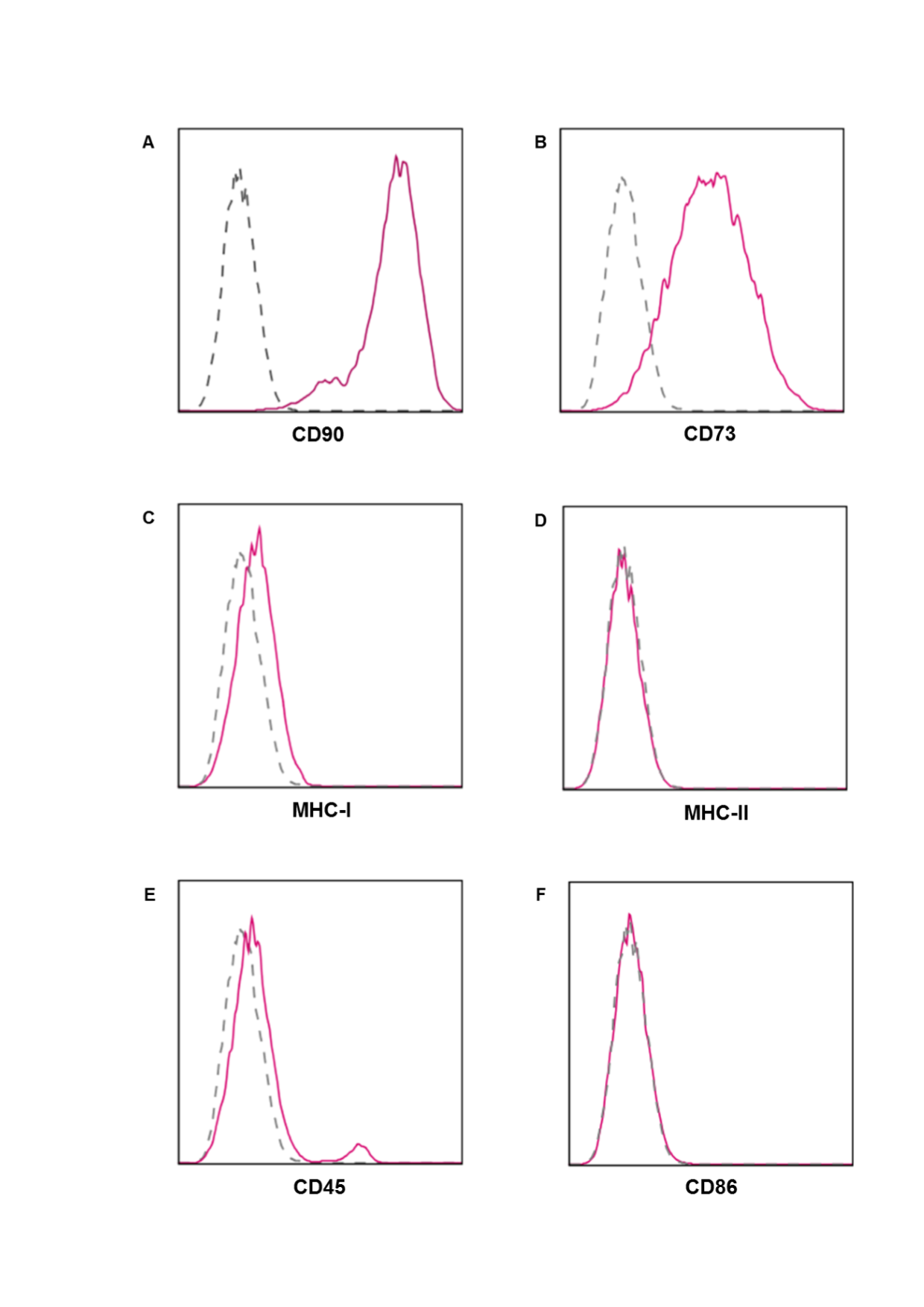

Supplement: Supplementary Figure 1 — MSC were characterized by flow cytometry using standard markers (A) CD90, (B) CD73, (C) MHC-I, (D) MHC-II, (E) CD45, and (F) CD86. [file Image_1.TIF]
